# Benchmarking Radiochromic EBT4 Film for Clinical Proton DosimetryRadiochromic Films for Proton Dosimetry

**DOI:** 10.1016/j.ijpt.2025.101294

**Published:** 2025-12-23

**Authors:** Robabeh Rahimi, Kuan Ling Chen, Jiajin Fan, Rao Khan

**Affiliations:** 1 University of Maryland School of Medicine, Baltimore, MD, USA; 2 Inova Schar Cancer Institute, Fairfax, VA, USA; 3 Department of Physics and Astronomy, Howard University, Washington, DC, USA

**Keywords:** Radiochromic film, Dosimetry, Proton beam, LET, Film under-response

## Abstract

**Purpose:**

This study presents an evaluation of the dosimetric performance of radiochromic EBT4 films for clinical proton therapy, benchmarked against the widely used EBT3 model. Key parameters assessed include dose-response behavior, energy dependence, sensitivity, reproducibility, temporal stability, and longitudinal and lateral linear energy transfer (LET) effects.

**Methods:**

EBT4 films from three independent batches and one batch of EBT3 films were irradiated using monoenergetic therapeutic clinical proton beams ranging from 70 to 225 MeV. Film irradiation was done at various depths in a solid water phantom, while doses from 0.25 to 20 Gy were delivered. Following scanning, the film responses were quantified as net optical density and calibrated with absolute dose measurements from a parallel plate ionization chamber. Temporal kinetics of net optical density were studied at various time points up to 120 hours post irradiation. Film dependency on LET of the proton beams was assessed through both lateral beam profiles and longitudinal depth-dependent analyses beyond pristine Bragg peaks.

**Results:**

EBT4 films exhibited a highly linear and reproducible dose-response (*R*² > 0.998), with minimal energy dependence (<3%) across 70 to 225 MeV proton energies. Compared to EBT3, EBT4 films showed an under-response of approximately 13%-20% at 10 Gy, depending on the batch, with a batch-to-batch variation of ∼8% observed between EBT4 films. Reproducibility between independent irradiations was within 1%, and sensitivity tests confirmed the ability to resolve dose variations as small as ±5% for doses as low as 0.5 Gy. The film’s optical density stabilized within 24 hours post-irradiation. LET-dependent response was observed in high-LET regions for EBT4 films, similar to EBT3 films.

**Conclusions:**

While EBT4 films exhibit relatively lower sensitivity compared to EBT3 and LET corrections remain necessary in high-gradient dose regions, EBT4 films are a promising candidate for routine and high-resolution proton dose measurements, provided that batch-specific calibration is performed.

## Introduction

Radiochromic films have become essential tools in radiation therapy dosimetry due to their high spatial resolution, near-tissue equivalence, and straightforward handling.^1^, ^2^ Early models such as Gafchromic EBT and EBT2 were initially developed for photon therapy and provided several advantages over traditional radiographic films, including wide dynamic range and the ability to be read without chemical processing.^3^, ^4^ Subsequent improvements led to the development of EBT3, featuring a symmetric film structure to minimize scanning artifacts and enhanced polymer chemistry, ensuring more consistent responses across both photon and proton beams. EBT3 rapidly became standard in clinical dosimetry for megavoltage photon and proton beams.^5^, ^6^, ^7^, ^8^, ^9^, ^10^, ^11^, ^12^

The most recent generation, Gafchromic EBT4, was designed to further enhance the performance and usability of its predecessor. According to the manufacturer and initial evaluations, EBT4 retains the favorable energy-independence and sensitivity characteristics of EBT3 while providing improvements in film uniformity, signal-to-noise ratio, and handling convenience.^13^, ^14^ Recent studies in photon modalities have confirmed EBT4′s improved image quality and enhanced agreement with planned dose distributions, highlighting its potential as a superior choice for clinical dosimetry applications.^15^, ^16^, ^17^, ^18^, ^19^ Khan et al recently provided a comprehensive characterization of the EBT4 film response for clinical X-ray therapy, establishing baseline dosimetric performance, minimum detectable dose limits, and optimal handling protocols.^20^ However, such evaluations have predominantly focused on photon beams, and a thorough characterization of EBT4 films under clinical proton beam conditions remains incomplete.

Proton therapy offers distinct advantages through the Bragg peak phenomenon, enabling precise dose localization at targeted depths and exceptional sparing of surrounding healthy tissues.^21^, ^22^ Nevertheless, accurate dosimetry in proton therapy is complicated by varying linear energy transfer (LET) along the beam path, especially near the Bragg peak and distal fall-off regions.^23^, ^24^, ^25^, ^26^ Radiochromic films are particularly susceptible to LET-dependent response variations, where films under-respond in high-LET regions, leading to dose underestimations if left uncorrected.^26^, ^27^ This effect has been well-characterized for EBT3 films, prompting several groups to develop empirical models and correction strategies to address this issue.^28^, ^29^ For instance, Anderson et al. reported a linear correlation between LET and under-response in EBT3 films, observing underestimations up to 15%-20% in distal high-LET regions.^29^ Similarly, Fiorini et al demonstrated dose under-response within spread-out Bragg peaks and proposed LET-correction methodologies to enhance measurement accuracy.^30^

Furthermore, while LET typically remains relatively constant within the primary treatment field, it may moderately increase at lateral distances due to contributions from slower, scattered protons.^28^, ^31^ In a 160 MeV proton beam, the dose-averaged LET at 5% of the central-axis dose (L_5_) was reported to be approximately 2 keV/μm higher than that on the central axis (L_cax_). This, however, was deemed insufficient to significantly alter biological outcomes such as relative biological effectiveness.

This study provides a systematic evaluation of EBT4 films for proton beam irradiations. Specifically, we investigate the dose response, energy dependence across proton energies ranging from 70 to 225 MeV, sensitivity to minor dose fluctuations, reproducibility, temporal evolution of the response post-irradiation, and LET dependency in both lateral beam profiles and depth-dependent scenarios. Our analysis utilizes multiple batches of EBT4 films and an EBT3 reference batch, with absolute dose measurements conducted using an ionization chamber for calibration and benchmarking purposes.

## Materials and methods

### Film materials and handling

Batches of Gafchromic EBT4 films (Ashland Inc., Bridgewater, NJ) labeled B1 (lot 07052202, expiration July 5, 2024), B2 (lot 07092301, expiration July 18, 2025), and B3 (lot 07202301, expiration July 19, 2025), along with one batch of Gafchromic EBT3 films labeled B4 (lot 09232202, expiration September 22, 2024), were used in this study. All films were stored and handled following the manufacturer’s and AAPM guidelines.^32^, ^33^, ^34^ Each film sheet was cut into approximately 40 × 40 mm² pieces, using a guillotine cutter to avoid damage to the polymer, under low light conditions and pre-scanned to measure baseline optical density. Orientation marks were applied to ensure consistent positioning during scanning. Films were scanned 48 hours post-irradiation to allow optical density stabilization unless specifically used for temporal response studies.

### Scanning and image analysis

Films were scanned using an Epson Expression 12000XL flatbed scanner (Epson America, Inc., Long Beach, CA) in transmission mode at 300 DPI (0.085 × 0.085 mm pixel size) and 48-bit RGB depth, and color corrections turned off, following recommended scanning protocols for Gafchromic films.^35^, ^36^ A fixed template ensured consistent film placement at the scanner center in landscape orientation. Net optical density (netOD) was calculated using the Beer-Lambert law:

$$\mathrm{netOD}=\log_{10}\left(\frac{I_{0}}{I}\right)$$

where $I_{0}$ and I represent the scanner signals for unexposed and exposed films, respectively. NetOD values were extracted individually for red, green, and blue channels using ImageJ version 1.53 m (National Institutes of Health, Bethesda, MD, USA) and custom MATLAB (MathWorks Inc., Natick, MA) scripts, analyzing the central film region to minimize edge effects. The uncertainty in netOD ($\sigma_{\mathrm{netOD}}$) was calculated as $\sigma_{\mathrm{netOD}}=\mathrm{netOD}\times \frac{1}{2.303}\sqrt{{\left(\frac{\sigma_{I_{0}}}{I_{0}}\right)}^{2}+{\left(\frac{\sigma_{I}}{I}\right)}^{2}}$.

### Irradiation setup

**Figure 1:**
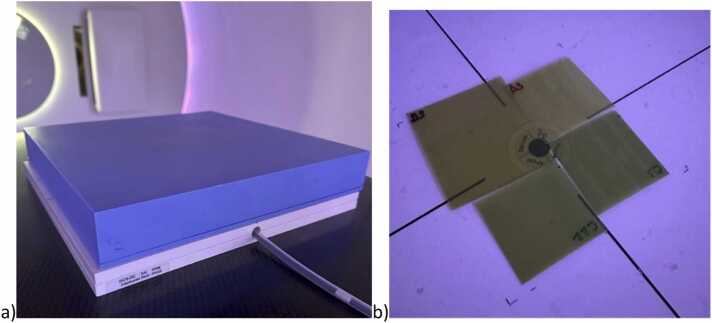
Experimental setup showing EBT3 and EBT4 radiochromic films positioned in phantom for various experimentations with different proton energies (a). Radiation dose was independently measured using a calibrated parallel plate ionization chamber (b).

Proton irradiations were conducted using an IBA ProteusPLUS pencil beam scanning system (IBA, Louvain-la-Neuve, Belgium). Films were positioned at specific water-equivalent depths (WEDs) in a phantom composed of RW3 solid water slabs (PTW, Freiburg, Germany). Absolute dose measurements were performed using a calibrated PPC05 parallel-plate ionization chamber (IBA Dosimetry, Schwarzenbruck, Germany) positioned directly beneath the films. Figure 1 shows the irradiation geometry in the proton room, film placement, and reference chamber setup. For all measurements iso was set to the surface. Water-equivalent thicknesses for all phantom components were calculated using depth-ionization data from a multi-layer ionization chamber, Zebra (IBA Dosimetry, Schwarzenbruck, Germany). All reported doses represent absorbed dose-to-water values measured at the film plane.

### Dose response calibration

Calibration curves were established by irradiating EBT4 films (batches B1-B3) and EBT3 films (batch B4) at 44 mm WED, corresponding to the plateau region of the 150 MeV beam, in order to avoid the Bragg peak and associated LET effects. A 100 × 100 mm² uniform field was delivered with doses ranging from 0.25 to 20 Gy (increments of 0.25 Gy up to 1 Gy, 1 Gy increments from 1 to 10 Gy, and 2 Gy increments thereafter).

NetOD was evaluated against chamber-measured dose, D, and a rational function of the form, $\mathrm{netOD}=\frac{a.D}{1+b.D^{n}}$, was independently fitted for each color channel. Fitting quality was evaluated using R² and root-mean-square error values below 10⁻³. The calibration function cannot be invertible in closed form; we instead chose to fit the empirical relationship $D=a\prime .\mathrm{netOD}+b\prime .\;{\mathrm{netOD}}^{n\prime }$, where an estimate of film doses from netOD was acquired.

### Energy dependence

To assess consistency across clinical proton energies, films were irradiated using monoenergetic beams of 70, 150, and 225 MeV. Film depths were selected within plateau regions (15 mm at 70 MeV, 44 mm at 150 MeV, and 84 mm at 225 MeV) to avoid Bragg peak effects. Each energy included three dose points: 0.5, 5, and 10 Gy. Responses were compared across batches and channels to evaluate energy-dependent variations.

### Reproducibility

Film reproducibility was assessed by irradiating EBT4 (B1-B3) and EBT3 (B4) films on two separate days under identical conditions (150 MeV, 44 mm WED, 0.5, 5, and 10 Gy). Ion chamber output was verified before each irradiation to ensure output consistency. NetOD results from both sessions were compared for inter-day consistency.

### Sensitivity (dose resolution)

Sensitivity (or film’s ability to resolve close dose values) was evaluated by irradiating the films at nominal doses of 0.5, 5, and 10 Gy and with ±5% variations (eg, at 5 Gy additional irradiations were 4.75 and 5.25 Gy). Dose-response linearity was assessed by comparing netOD variations against measurement noise to determine the films’ ability to distinguish small dose changes.

### Temporal response

Temporal stability was investigated by irradiating films at doses of 1, 5, and 10 Gy (at 150 and 225 MeV) and scanning at intervals: 0, 0.5, 1, 2, 4, 8, 24, 48, and 120 hours post-irradiation. A control film was scanned at each time point to correct for scanner response variation. Optical density evolution was modeled using a biexponential function: $\mathrm{netOD}(t)={\mathrm{netOD}}^{\infty }-(C_{1}e^{-\frac{t}{T_{1}}}+C_{2}e^{-\frac{t}{T_{2}}})$. The exponential fitting was done by using a nonlinear fitting Levenberg Marquardt algorithm.^37^ ${\mathrm{netOD}}^{\infty }$represents the saturation value of the net optical density, $C_{i}$, $T_{i}$ (i = 1, 2) parameters were obtained by optimization. The biexponential fits optimization was continued until the root mean-square error between the model and the measured data was below 10^−3^ in each case.

The differential growth rate, given in %/h, $\mathrm{Differential growth rate}\left(\frac{\%}{h}\right)=-\frac{1}{{\mathrm{netOD}}^{48h}}\frac{\partial \mathrm{netOD}}{\partial t}\times 100$ for each color is defined by numerically differentiating the net optical density of each color with time and normalizing to the corresponding net optical density at 48 hours.^11^

Numerical differentiation for differential growth rate was selected to mitigate the risks of overfitting or underfitting associated with biexponential models, which may not consistently capture the underlying dynamics across all scenarios. While a triexponential model has been justifiable in certain cases, choosing a specific functional form introduces potential bias and may fail to reflect the true experimental behavior. Despite the known limitations of numerical differentiation, particularly its sensitivity to noise in experimental data, it offers a model-independent approach that avoids assumptions about the functional structure. The differential growth rate for all the films is computed and included in the figures.

### LET dependence—longitudinal profile (Bragg peak)

Films were irradiated with a pristine 150 MeV proton beam at depth intervals of 1 mm spanning the Bragg peak and distal fall-off regions. Films were placed in solid water slabs and positioned directly above the PPC05 chamber, with depth steps of 1 mm spanning the Bragg peak and distal fall-off regions, to ensure consistent alignment between chamber and film measurements. LET dependencies were compared for EBT4 and EBT3 films.

### LET dependence—lateral profile

To evaluate LET effects, EBT4 film responses were compared to PPC05 parallel-plate ionization chamber measurements across the lateral penumbra of a uniform 100 × 100 mm² proton field at two water-equivalent depths of 44 mm and 150 mm. The PPC05 chamber was oriented perpendicular (90°) to the beam axis in the water tank, reducing the effective detector dimension in the lateral gradient direction to approximately 1 mm and enabling high-resolution point-by-point measurements across the penumbra.

While this detector orientation introduces depth-wise volume averaging, measurements were performed at a fixed depth, and the chamber was translated only laterally. Film measurements at the central axis were normalized to the PPC05 reading at the central axis, and both film and chamber readings at lateral positions were analyzed relative to this normalized value. Consequently, depth-wise volume averaging was identical for all lateral positions and acted as a common-mode contribution. Volume averaging in the penumbra is unavoidable for any finite-sized detector, the chosen chamber orientation minimized its impact on the measured lateral dose profiles.

Film-derived dose profiles were compared with chamber data, normalized to the central axis, and analyzed for dose response near the field edges to extract the lateral LET response of the film.

## Results

The results corresponding to each experiment are presented as follows.

### Dose dependency

**Figure 2:**
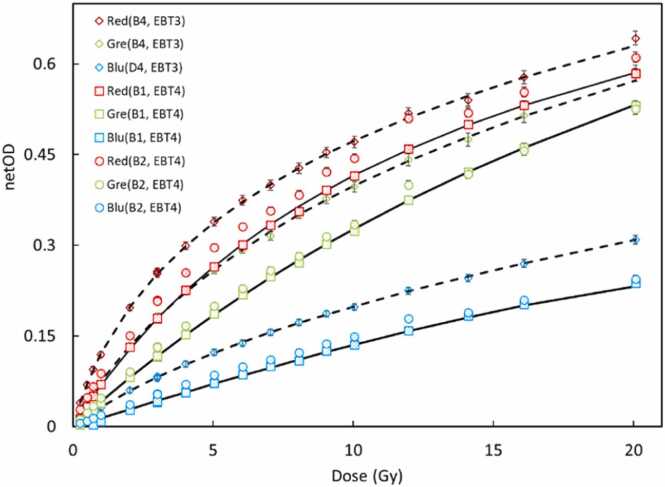
Dose-response calibration curves for EBT3 and EBT4 films irradiated with 150 MeV protons, showing netOD as a function of absorbed dose for red, green, and blue channels across multiple batches. EBT4 data are shown for two batches (B1 and B2), and EBT3 data are shown for the B4 batch. Dashed line fits are displayed for EBT3, whereas solid lines are shown for B1 batch, fit for B2 are not shown to improve readability.

Calibration curves for EBT4 and EBT3 films irradiated with 150 MeV protons were generated over a dose range from 0.25 to 20 Gy. As shown in Figure 2, the netOD increased monotonically with dose for all three color channels (red, green, and blue). Consistent with prior reports, the red channel demonstrated the highest sensitivity, followed by the green and blue channels.

The fitting parameters, *a*, *b*, and *n*, for a rational function dose–response model, $\mathrm{netOD}=\frac{a.D}{1+b.D^{n}}$ along with corresponding coefficients of determination (*R*²), are provided in Table 1. All channel fits resulted in high *R*² values (>0.998), indicating excellent agreement between measured data and the model. Although the functional relationship was consistent across film types and batches, absolute sensitivities varied significantly.

**Table 1:** Fitting parameters (*a, b, n*) for the rational function model used to describe the dose–response relationship of $\mathrm{netOD}=\frac{a.D}{1+b.D^{n}}$ derived for each color channel of EBT3 and EBT4 films.

Film	Color (Batch)	*a*	*b*	*n*	*R*^2^
EBT4	Red (B1)	0.0740	0.0842	0.9684	0.9998
	Green (B1)	0.0418	0.0258	1.0355	0.9994
	Blue (B1)	0.0142	0.0004	2.1124	0.9998
	Red (B2)	0.1084	0.2339	0.7978	0.9997
	Green (B2)	0.0532	0.0959	0.7919	0.9995
	Blue (B2)	0.0205	0.0593	0.8161	0.9997
EBT3	Red (B4)	0.1844	0.5289	0.7403	0.9989
	Green (B4)	0.0912	0.2252	0.7597	0.9997
	Blue (B4)	0.0393	0.2132	0.6624	0.9998

The results indicate high goodness of fit (*R² > 0.998 for all fits), with variations in absolute sensitivity across film batches and channels, supporting the need for batch-specific calibration.*

**Table 2:** Deviation from PPC05 reading in the field center, measured at lateral distances 51, 55, and 58 mm at 44 mm depth; and 54, 59 and 64 mm at 150 mm depth from the center in a 100 x 100 mm² field size.

44 mm depth	Relative dose w.r.t central axis		
Lateral position	PPC05	B2	B3
L_51 mm_	52.6%	51.7%	54.5%
L_55 mm_	18.7%	16.1%	18.9%
L_58 mm_	6.0%	4.8%	7.9%

150 mm depth	Relative dose w.r.t central axis		

Lateral position	PPC05	B2	B3

L_54 mm_	51.7%	56.0%	62.5%
L_59 mm_	19.5%	22.7%	29.2%
L_64 mm_	5.6%	5.56%	8.5%

A notable observation is the batch-to-batch variability within EBT4 films. At 10 Gy dose level, variations of about 12.2%, 15.2%, and 15.0% were observed for the red, green, and blue channels, respectively, between the B1 and B2 batches. Therefore, a batch-specific calibration is necessary when employing EBT4 films in clinical dosimetry.

Comparing overall sensitivity to EBT3 (batch B4), EBT4 films consistently exhibited a lower netOD response across all color channels. Specifically, red, green, and blue channels batch B2 were 6.3%, 18.9% and 22.7%, lower than batch B4, and red, green and blue channels batch B1 were 12.5%, 10.8%, and 9.1%, lower than batch B4. These results corroborate prior observations regarding the reduced sensitivity of EBT4 relative to EBT3.^11^ Despite variations in absolute sensitivity, the overall shape of the dose–response curves remained consistent, supporting the reliability of the applied model and the feasibility of relative dosimetry following appropriate calibration.

### Response to proton energy variation

**Figure 3:**
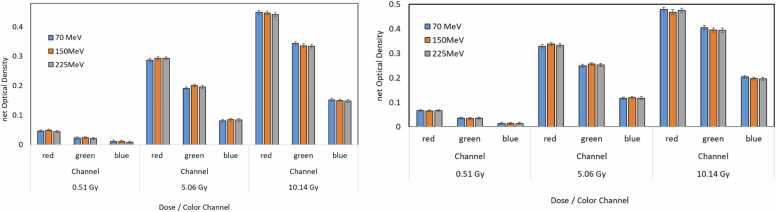
Energy dependency of EBT4 (left) and EBT3 (right) films irradiated with proton beams of 70, 150, and 225 MeV at doses of 0.5, 5, and 10 Gy. Bar plots show the netOD for red, green, and blue channels at each energy and dose level. For both film types, the variation in netOD across proton energies is minimal and within experimental uncertainty, indicating no significant energy dependence within the 70-225 MeV clinical range.

Figure 3 demonstrates the energy dependency of EBT4 and EBT3 films across 70, 150, and 225 MeV beams at doses of 0.5, 5, and 10 Gy. Both film types showed minimal variation in netOD with energy at a fixed dose, with red channel netOD at 5 Gy being ∼0.30 at 70 MeV, ∼0.32 at 150 MeV, and ∼0.33 at 225 MeV. This variation is within experimental uncertainty, confirming no significant energy dependency in EBT4 across clinically relevant proton energies.

### Film reproducibility

Reproducibility was assessed via independent irradiations performed on separate days at doses of 0.5, 5, and 10 Gy using the 150 MeV beam. Films irradiated on Day 1 and Day 2, were each scanned 48 hours post-irradiation. Variations in netOD of less than 1% for red and green channels across all doses were observed (Supplementary Material Figure S1). For instance, red channel netOD at 10 Gy was 0.4426 on Day 1 and 0.4403 on Day 2. The low variability confirms the high reproducibility of EBT4 film measurements under consistent setup conditions.

### Film sensitivity

Sensitivity was evaluated by applying ±5% dose variations around three nominal dose levels: approximately 0.5, 5, and 10 Gy (0.476, 0.505, 0.523 Gy; 4.818, 5.071, 5.329 Gy; 9.625 Gy, 10.142 Gy, 10.659 Gy) across all color channels (Supplementary Material Figure S2). Sensitivity assessment was performed by comparing the measured netOD values with the predicted netODs derived from the dose–response equation presented in Section 2.4, $\mathrm{netOD}=\frac{a.D}{1+b.D^{n}}$, using the fitted parameters listed in Table 1. The full comparison, including predicted netODs, measured netODs, and percentage deviations for all color channels of EBT3 and EBT4, is provided in Supplementary Material Table S1.

The red channel has demonstrated the highest sensitivity at the low-dose region (≈0.5 Gy), with deviations between measured and predicted netOD were generally within ±3% for EBT4 and ±8% for EBT3. For intermediate doses (≈5 Gy), the deviations were around ±3% for both EBT4 and EBT3. For the high-dose region (≈10 Gy), the agreement remained strong, with deviations reduced to ±1.5% for EBT4 and EBT3. From these results, we can conclude that both EBT3 and EBT4 films exhibit dose-dependent sensitivity that matches acceptedly with the fitted dose–response model, with improved agreement at mid-to-high doses.

### Temporal response

**Figure 4:**
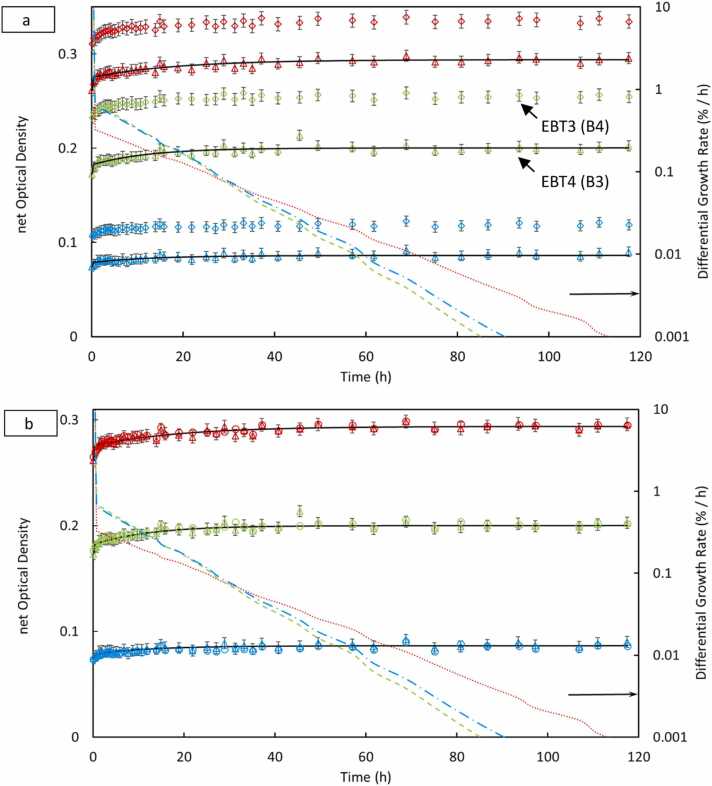
Biexponential fit and differential growth for EBT4 and EBT3 films, for 70 MeV at 5 Gy dose level. Arrow shows the right ordinate for the differential growth rate data for the corresponding three colors of EBT4 (B3) batch. (a) shows EBT4 (B3) vs EBT3 (B4), (b) shows the EBT4 inter-batch comparison, B2 (∆) vs B3 (o).

**Figure 5:**
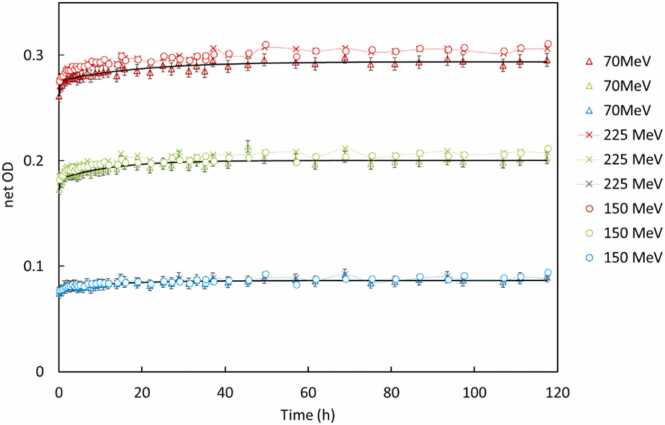
Temporal response of netOD at 5 Gy (EBT4-B1, ∆; B3, x; and EBT3, B4, o) for all three proton energies (70, 150, and 225 MeV). The netOD progression for each color channel is plotted along with corresponding bi-exponential fits (solid dark line). The overlapping curves indicate negligible influence of proton energy on the time-dependent film darkening, validating the use of a single time-correction model or a universal scan delay protocol across different beam energies.

The temporal evolution of netOD post-irradiation was monitored for both EBT4 and EBT3 films over 120 hours across the three proton energies (70, 150, and 225 MeV) and at dose levels of 1, 5, and 10 Gy. Figure 4, Figure 5 and S3-S4 (Supplementary Material) illustrate the netOD kinetics for each energy and dose combination, plotted for EBT4 and EBT3 films. Both film types exhibited rapid polymerization during the initial hours after irradiation, characterized by a sharp increase in netOD within approximately the first 6-12 hours. The optical density growth rate gradually decreased thereafter, approaching stabilization around 24 to 48 hours. This general pattern was consistent across all proton energies and dose levels tested. We can identify the plateau region of color growth as the point where the differential growth rate falls below 0.05% per hour.^20^ While some polymerization and color development may still occur at this threshold, it does not significantly affect the net optical density and, therefore, has minimal impact on dose estimation.

Both EBT3 and EBT4 films show minimal sensitivity to continued color growth once the differential rate drops below this threshold. This stabilization corresponds to the consistent net optical density trends observed across low, medium, and high dose ranges.

Our results suggest that scanning films for between 40 and 60 hours post-irradiation provides a reliable window where the dose measurements are stable and less affected by post-exposure changes.

At 70 MeV (Figure 4a), EBT4 films reached approximately 95% of their final netOD within the first 2 hours, slightly faster than the corresponding EBT3 response. Figure 4b shows inter-batch consistency in temporal buildup response. Similar rapid initial growth trends were observed at higher energies and doses (Figures S3 and S4 in Supplementary Material), with no evidence of signal decay or instability observed up to 120 hours post-irradiation, confirming excellent long-term temporal stability.

The differential growth rate analysis further confirmed these observations. Differential growth rates indicated an exponential reduction in polymerization rate following irradiation. All channels, particularly red and green, demonstrated the highest polymerization rates immediately post-irradiation, which rapidly declined below 0.1%/h after 24 hours. The blue channel, being less sensitive, exhibited lower overall differential growth rates and flatter netOD curves.

Comparatively, EBT4 exhibited smoother netOD stabilization trends and a faster decline in differential growth rate compared to EBT3, particularly notable at higher doses (5 and 10 Gy) and energies. At the highest tested dose (10 Gy), EBT3 films showed larger variability in growth rates, particularly in the red and green channels, suggesting slightly less consistent temporal stabilization than EBT4. Across the energies tested, no significant differences were noted in stabilization time.

Figure 5 summarizes the mean netOD growth for EBT4 films at 5 Gy across energies, fitted using a bi-exponential growth model. The curves demonstrate that temporal behavior is largely independent of proton energy, with minor observed differences likely attributable to statistical fluctuations or slight batch variations. Collectively, these findings validate the practicality of adopting a standardized 48-hour post-irradiation scan time in clinical workflows, ensuring minimized readout uncertainties and robust dose evaluation using both EBT4 and EBT3 films.

### LET dependency—pristine Bragg Peak

**Figure 6:**
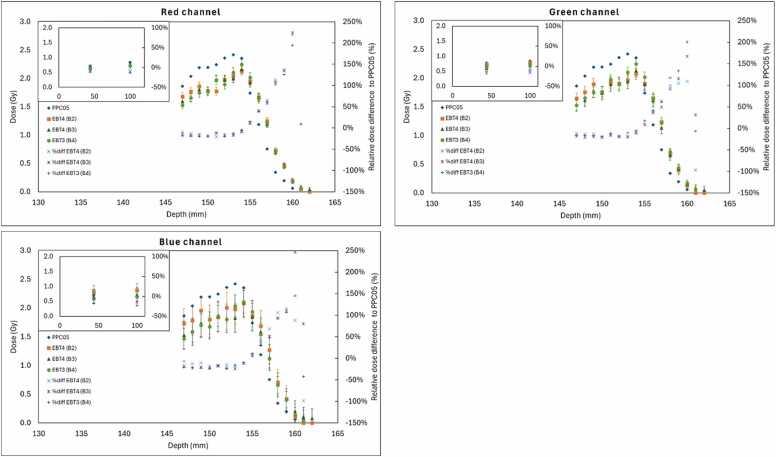
Integrated depth-dose profiles (IDDs) at 150 MeV comparing EBT4 film batches B2 and B3, and EBT3 film batch B4, to PPC05 ion chamber measurements in red (top left), green (top right), and blue (bottom) color channels. All channels show good agreement in the entrance and plateau regions, while the films, EBT4 and EBT3, underestimate dose relative to the chamber near the Bragg peak. Beyond the Brag peak region, and because of the dose gradient, it was practically difficult to match the films’ readings with the ion chamber; however, EBT4 films show similar responds as EBT3 film in this region. The relative dose differences to PPC05 ion chamber doses were also plotted $\left((\mathrm{film dose}/\mathrm{PPC}05\mathrm{dose})-1\right)*100\%$.

LET effects at the Bragg peak were evaluated by placing EBT4 films along the central beam axis and comparing their measured dose to PPC05 ionization chamber readings for a 150 MeV proton beam. Figure 6 presents the integrated depth-dose profiles for EBT4 batches B2 and B3 red, green, and blue channels, alongside the ionization chamber data and EBT3 batch B4.

Across all color channels, EBT4 films generally agree with PPC05 in the entrance and mid-depth plateau regions. However, approaching the Bragg peak, the film-predicted dose consistently drops off more steeply than the chamber-based dose. Compared the EBT4 film readings, B2 and B3, and the EBT3 film reading, B4, with ion chamber readings, which have similar under-responses. After the Bragg peak, the dose gradient is large, and matching the films’ reading with PPC05 was not practically possible; however, EBT4 films showed similar responds and the EBT3 film, beyond the Bragg peak region too.

### LET dependency – lateral profile

LET dependent under-response was assessed by comparing EBT4 film responses to PPC05 ionization chamber measurements across the lateral penumbra of a 100 × 100 mm² proton field at two WEDs: 44 and 150 mm. The PPC05 chamber was oriented perpendicular (90°) to the beam axis and translated laterally in 1-mm increments. This approach allowed high spatial resolution measurement for dose at field edges.

**Figure 7:**
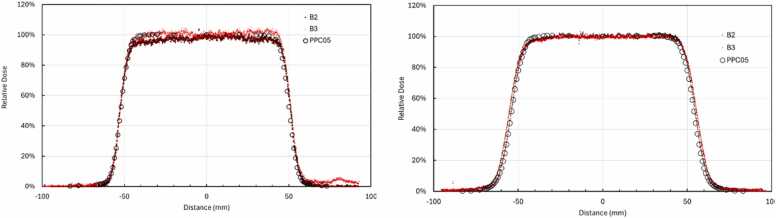
Lateral dose profiles comparing EBT4 film (batches B2 and B3) to PPC05 ion chamber measurements at depths of 44 mm (left) and 150 mm (right) in a 100 × 100 mm² proton field.

As shown in Figure 7, both EBT4 batches (B2 and B3) closely matched the PPC05 chamber readings in the central high-dose region of the beam and in the lateral fall-off regions. No significant depth-dependent variation in the lateral LET response was observed between the 44 and 150 mm depths, aligning with Monte Carlo simulation results.^17^

## Discussions

This study presents a comprehensive assessment of the radiological performance of Gafchromic EBT4 film for proton therapy dosimetry, benchmarked against the widely used EBT3 model. Comparative evaluations have been done across multiple key domains, including dose-response behavior, batch consistency, energy dependence, temporal development, sensitivity, and LET response.

EBT4 films exhibited a strong and reproducible dose-response relationship, with calibration curves fitting well to a power law model (*R*² > 0.998). Across multiple batches, EBT4 demonstrated slightly lower netOD per unit dose compared to EBT3, which is consistent with prior observations.^11^ However, the inter-batch variation was observed, reinforcing the necessity of individual batch calibration.

Qualitatively, across all tests, the red channel consistently provided the highest netOD and lowest noise, making it the most suitable for dosimetry. Green channels exhibited moderate sensitivity and low noise, while the blue channel had the lowest signal but acceptable performance for certain applications. To illustrate this with a specific example, based on the dose-response data in Figure 2, the mean noise-to-signal ratios were 2.57% for the red channel, 4.07% for the green channel, and 9.77% for the blue channel, confirming the superior performance of the red channel.

A key dosimetric attribute of EBT4 is its minimal dependence on proton beam energy across the 70 to 225 MeV clinical range, excluding high-LET distal Bragg peak conditions. Our data revealed less than 3% variation in film response across this spectrum, which falls within acceptable clinical tolerances and confirms that EBT4, like EBT3, is effectively water-equivalent in this energy range. In distal high-LET regions, however, under-response is observed, and LET corrections are required. This energy independence permits EBT4 use across mixed-energy treatment beams without introducing significant uncertainty, provided LET corrections are properly addressed.

Post-irradiation darkening of EBT4 films followed a characteristic growth pattern, with >90% of final netOD reached within 6-8 hours and full stabilization by 24 hours. At 48 hours the EBT4 kinetics mirror those of EBT3, validating that readout timing protocols developed for EBT3 can be directly applied to EBT4. The slightly faster polymerization kinetics observed in some batches of EBT4 at low doses may reflect subtle differences in monomer composition or layer thickness but may not introduce meaningful changes in protocol requirements. Correction factors derived from early timepoint netOD measurements may be used if early readout is warranted.

Daily reproducibility tests showed that EBT4 films maintain high precision across repeated measurements, with dose differences under 1% for doses above 1 Gy. For very low doses (eg, 0.5 Gy), slightly higher variability (∼10%-15%) was observed in the blue channel due to inherently low signal levels. However, this behavior is not unique to EBT4 and is typically mitigated in clinical workflows by utilizing red channel or multichannel analysis. Film positioning and orientation in scan and irradiation were tightly controlled in our study to minimize lateral response artifact by using small films and reproducible location of ROI. Lateral response artifact remains an active area of investigation for planar dose measurements.

Like EBT3, EBT4 exhibited LET-dependent under-response at regions of high proton LET, particularly at the Bragg peak. The magnitude and shape of this under-response were nearly similar between the two models. While EBT4′s LET-dependent under-response behavior imposes limits on its direct use for absolute dose in high-LET regions, its accurate spatial mapping of dose distributions remains valuable for QA, especially in combination with chamber or simulation data.

EBT4 and EBT3 films exhibited similar behavior across various tests, slightly lower dose response for EBT4, energy independence, reproducibility, and LET sensitivity. The differences between film types were minimal, with EBT4 showing slightly faster temporal response and similar LET under-response patterns. As EBT4 adoption increases and EBT3 production tapers, this validation ensures continuity in film-based dosimetry, providing clinicians with a robust and up-to-date tool for high-precision verification in proton therapy.

## Conclusion

This study provides a comprehensive assessment of Gafchromic EBT4 film for proton beam dosimetry, including dose response, energy and LET dependence, reproducibility, and temporal stability. Compared to EBT3, EBT4 showed slightly lower netOD response (6%-23% lower depending on batch and channel) but maintained consistent, predictable behavior with good reproducibility and no energy dependence across 70 to 225 MeV. LET-induced under-response was observed and should be corrected using correction factors if EBT4 film is to be used in varying LET regions. EBT4 introduced no new limitations; therefore, despite its lower sensitivity compared to EBT3, EBT4 can be used for clinical proton film dosimetry with proper calibration.

This study was conducted under clinical dose-rate conditions. Dose-rate dependence under ultra-high-dose-rate (FLASH) conditions was not evaluated and remains an important subject for future investigation. For ionization chambers, we note that the PPC05 (air-filled, parallel-plate chamber) exhibits minimal but measurable LET-related recombination effects, typically within ∼1% in the mid-energy plateau region and up to ∼2% near the distal high-LET Bragg peak.^38^ These corrections are small in the clinical context but should be acknowledged when high precision is required.

## CRediT authorship contribution statement

RR, KC, and RK conceived and designed the study. RR and KC performed the proton irradiation at ISCI, and RK and KC scanned the films and curated the data. All authors contributed to data analysis, with JF providing additional review of results and discussion. RR, KC, and RK drafted the initial manuscript, and all authors participated in editing. RK and JF supervised the project.

## Declaration of Conflicts of Interest

The authors declare that they have no known competing financial interests or personal relationships that could have appeared to influence the work reported in this paper.

## Footnotes

Appendix A Supplementary data associated with this article can be found in the online version at doi:10.1016/j.ijpt.2025.101294.
