# Supplementary material for: Benchmarking Radiochromic EBT4 Film for Clinical Proton DosimetryRadiochromic Films for Proton Dosimetry
Source: Int J Part Ther. 2025 Dec 23;19:101294. doi: 10.1016/j.ijpt.2025.101294 (PMC12818114; doi:10.1016/j.ijpt.2025.101294)
Supplement: Supplementary file 1 — Supplementary material [file mmc1.docx]

**Supplementary material**

**Benchmarking Radiochromic EBT4 film for Clinical Proton Dosimetry**

Figure S1 shows variations in netOD of less than 1% for red and green channels across all doses. For instance, red channel netOD at 10 Gy was 0.4426 on Day 1 and 0.4403 on Day 2. The low variability confirms the reproducibility of EBT4 film measurements under consistent setup conditions.


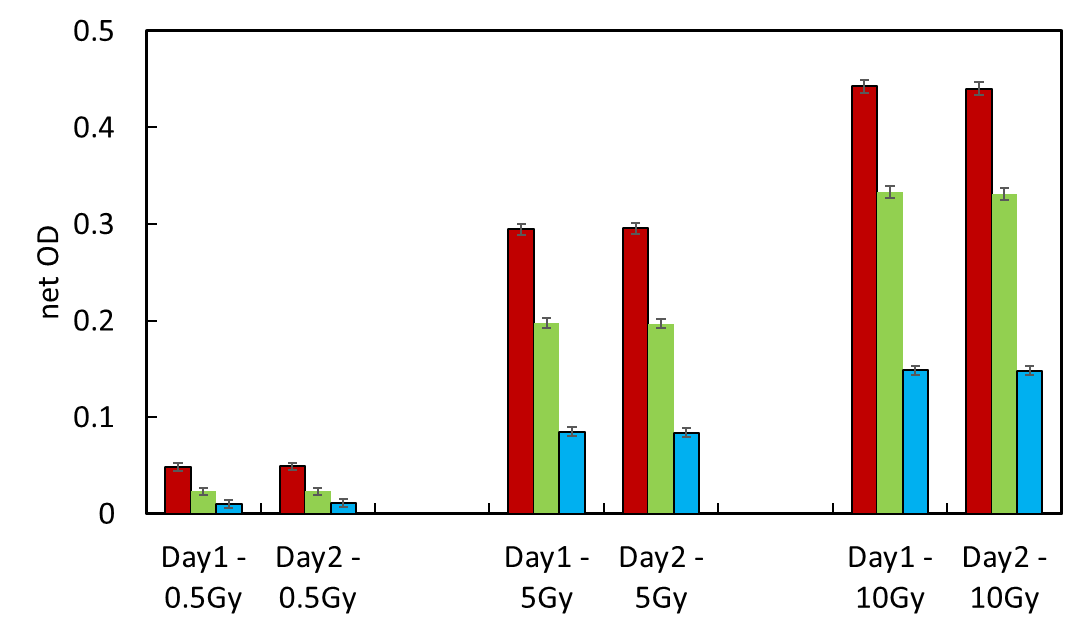


Figure S1 Day-to-day reproducibility of EBT4 film response at 0.5 Gy, 5 Gy, and 10 Gy for red, green, and blue channels. Films were irradiated on two separate days using a 150 MeV proton beam. The measured netODs show small variation between days, with differences typically <1% for red and green channels, confirming high reproducibility under consistent irradiation and scanning conditions.

Figure S2 and Table S1 demonstrate the sensitivity of the films. Figure S2 shows that films, EBT4 (B2) and EBT3 (B4), are capable of detecting small dose deviations to the nominal dose levels. Table S1 quantifies this performance by reporting the differences between the calculated nominal doses and the measured net optical density values.

| 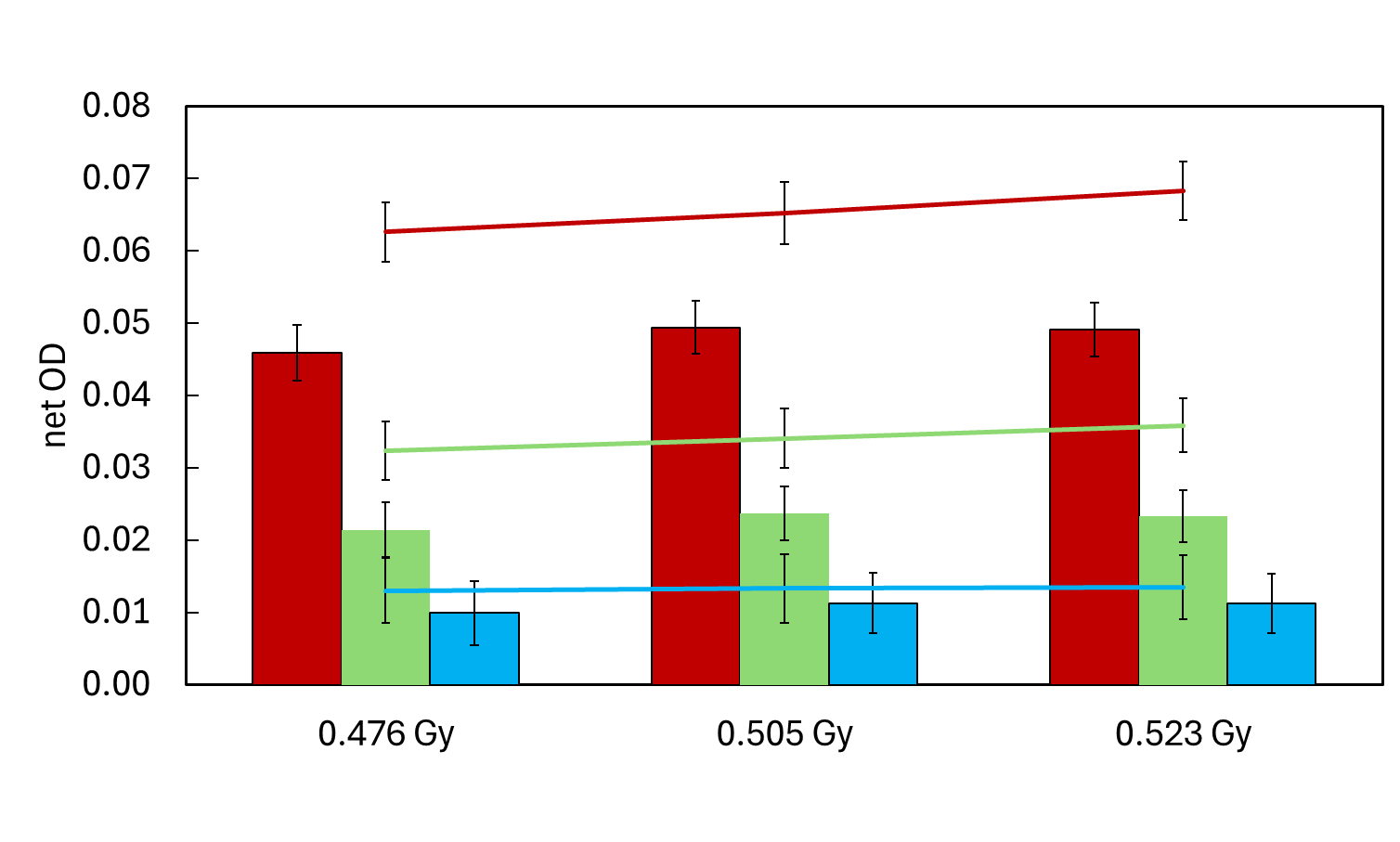 | 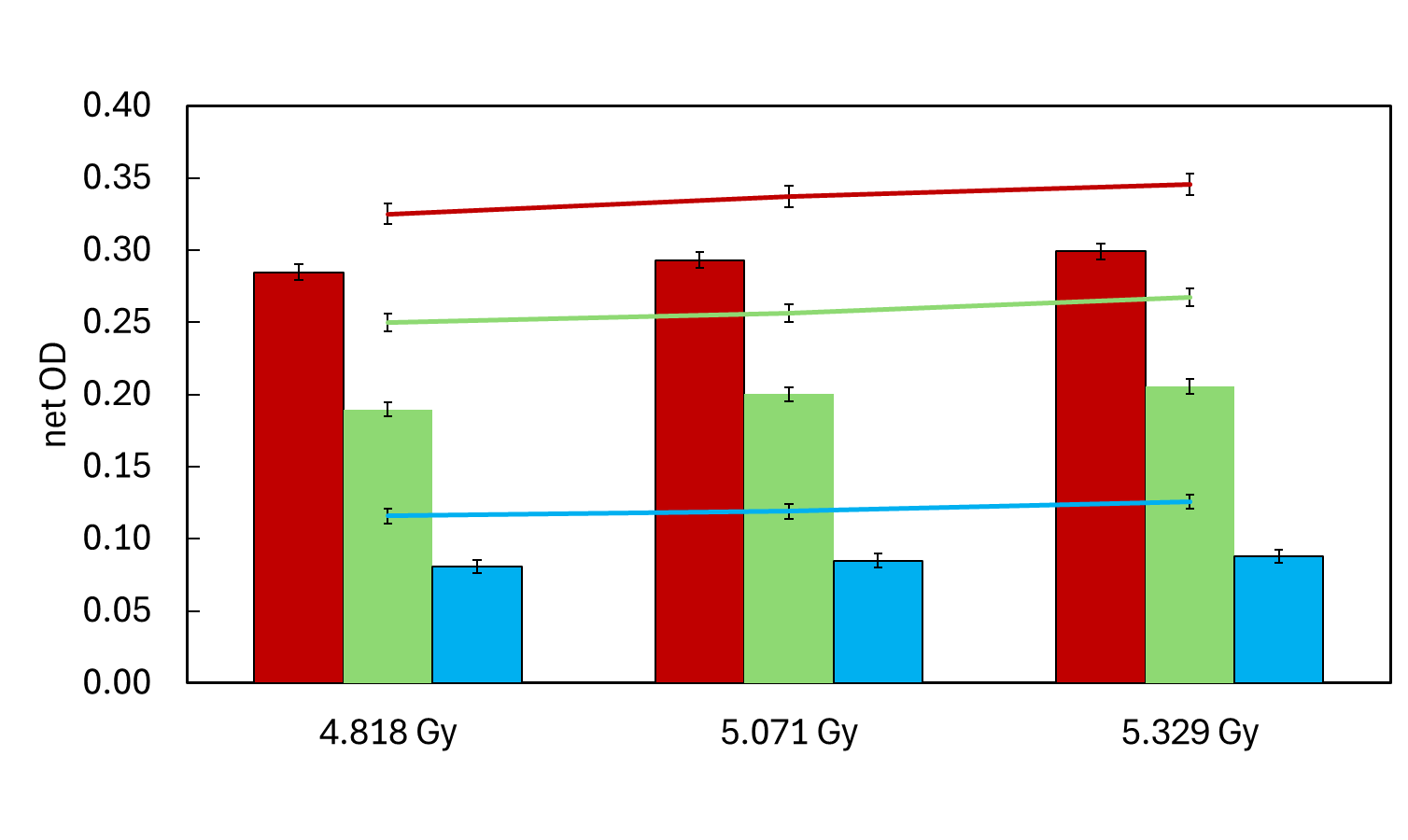 |
| --- | --- |
| 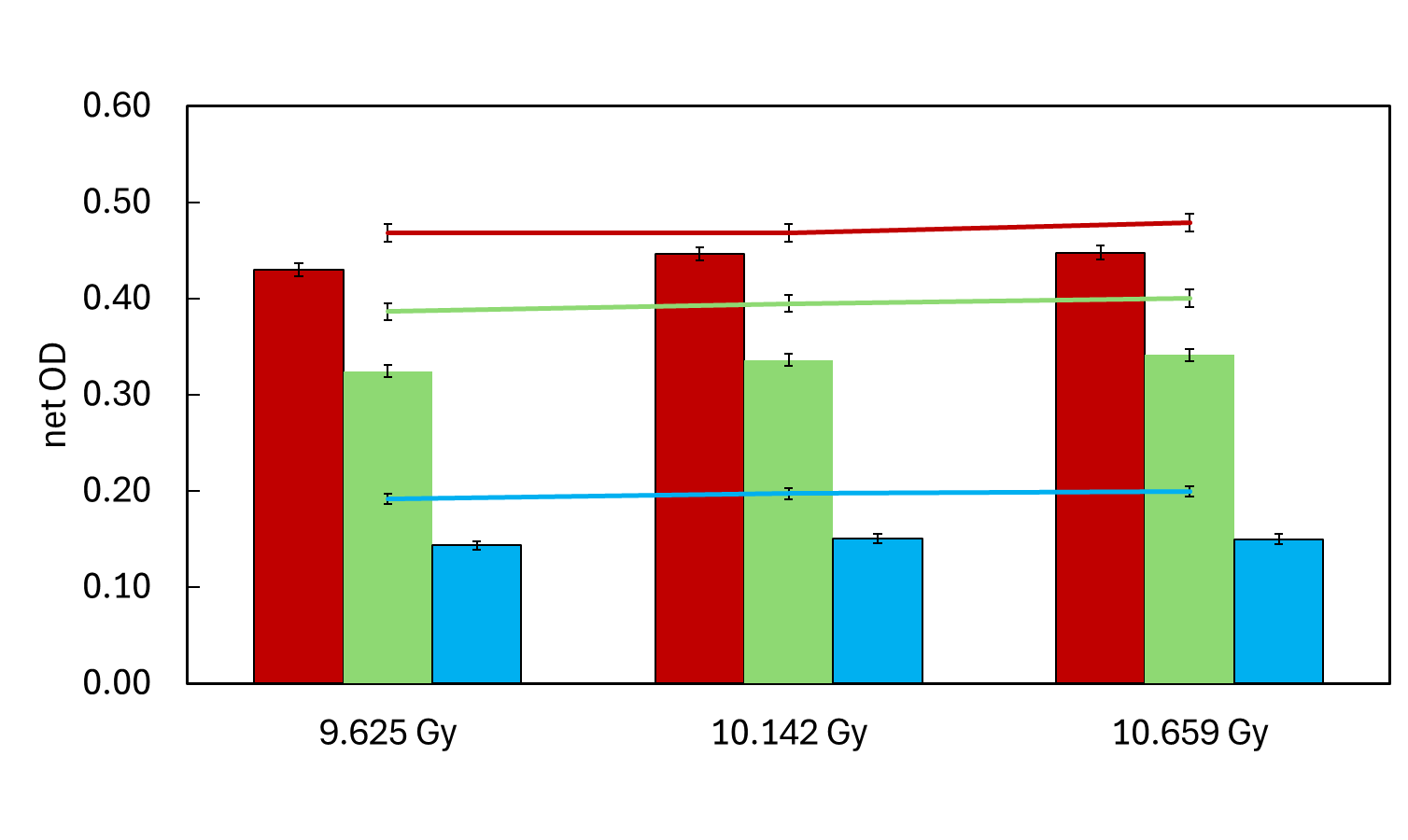 | Figure S2 Films sensitivity of EBT4 (shown as bars) and EBT3 (shown as line plot) evaluated by introducing ±5% dose variations around the nominal dose points (0.5 Gy, 5 Gy, and 10 Gy). Both films show measurable changes in net optical density in response to small dose perturbations, demonstrating their ability to detect subtle dose differences. The accuracy of the detected changes is assessed by comparing the measured values with the expected values calculated in Table S1. |

Table S1 Expected netOD values calculated from the rational function model $netOD=\frac{a.D}{1+b.D^{n}}$ using the fitted parameters, compared with the measured netOD values for the sensitivity test doses (0.5 Gy, 5 Gy, and 10 Gy) and for ±5% dose variations around those levels. The table reports the predicted netOD, the measured netOD, and the percentage difference between them for the red, green, and blue channels of EBT4 (B2) and EBT3 (B4) films.

Figures S3 and S4 show the temporal response of EBT4 and EBT3 films exposed to 150 MeV (S3) and 225 MeV (S4) protons at 1 Gy, 5 Gy, and 10 Gy. The rapid darkening phase occurs within the first 6–12 hours, followed by a plateauing trend thereafter. Across all doses, EBT4 and EBT3 exhibit similar kinetic trends and stabilization characteristics. The arrows show the right ordinate for the differential growth rate data for the corresponding three colors of EBT4 and EBT3 films.

| **EBT4** | | **EBT3** |  |
| --- | --- | --- | --- |
| 1 Gy  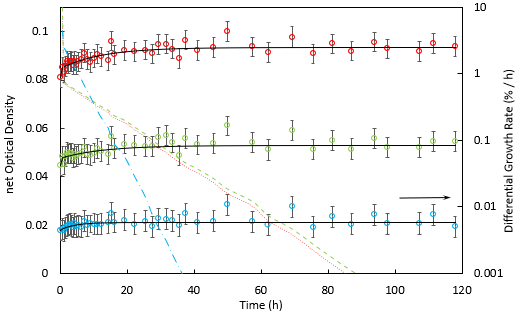 | | 1 Gy  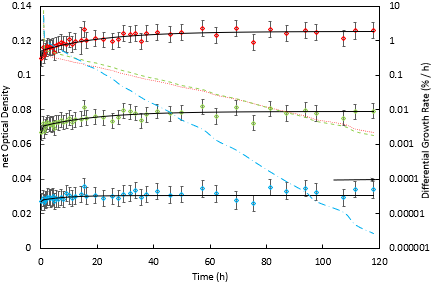 |  |
| 5 Gy  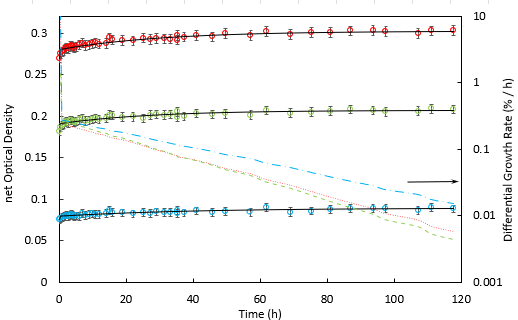 | | 5 Gy  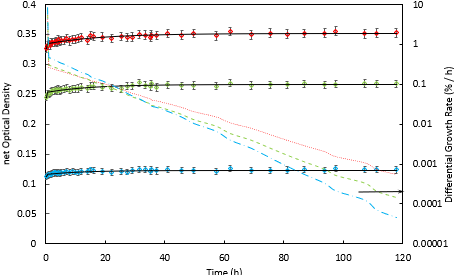 |  |
| 10 Gy  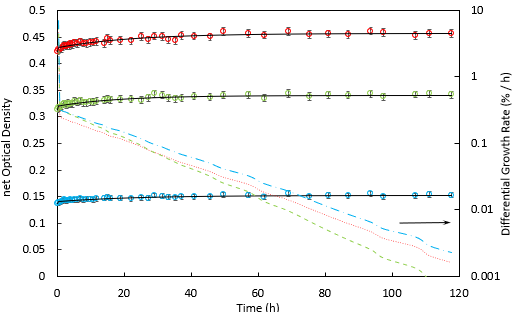 | | 10 Gy  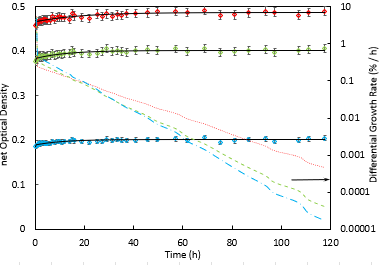 |  |
| Figure S3 Temporal response of EBT4 (left column) and EBT3 (right column) films exposed to 150 MeV protons at 1 Gy, 5 Gy, and 10 Gy. The rapid darkening phase is seen within the first 6–12 hours, with plateauing trends thereafter. Across all doses, EBT4 and EBT3 demonstrate similar kinetics and stabilization behavior. The arrows show the right ordinate for the differential growth rate data for corresponding three colors of EBT4 (left) and EBT3 (right) films. | | |  |
| **EBT4** | **EBT3** | | |
| 1 Gy  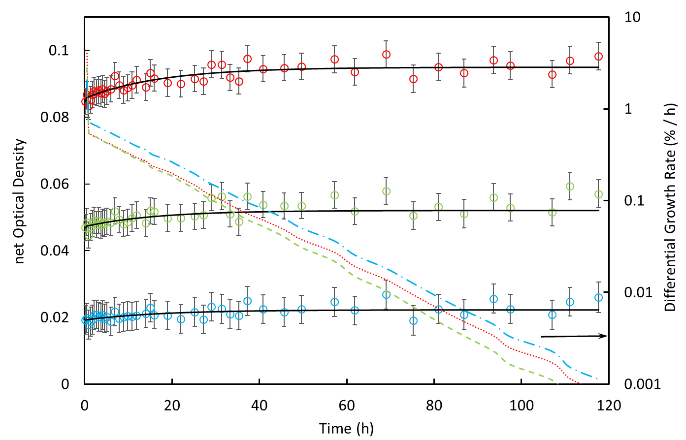 | 1 Gy  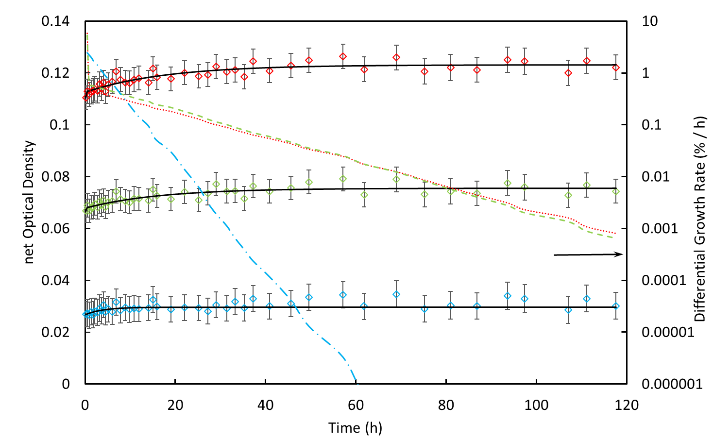 | | |
| 5 Gy  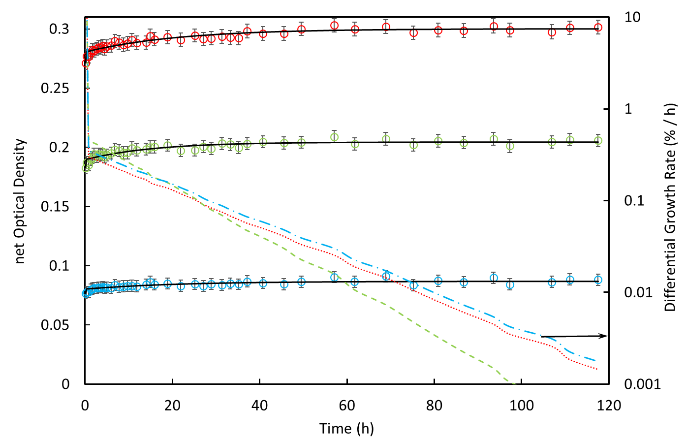 | 5 Gy  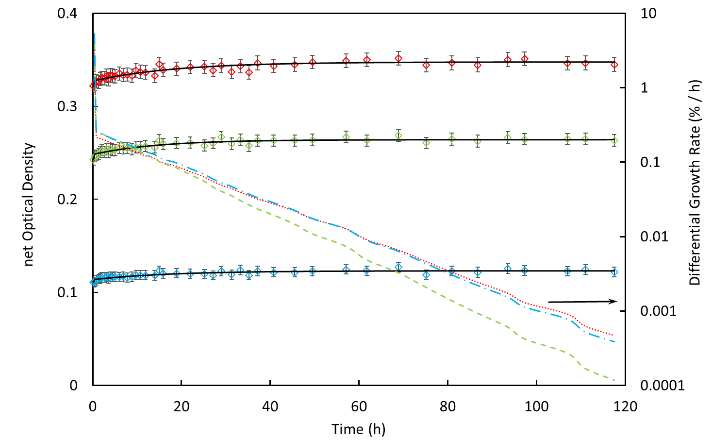 | | |
| 10 Gy  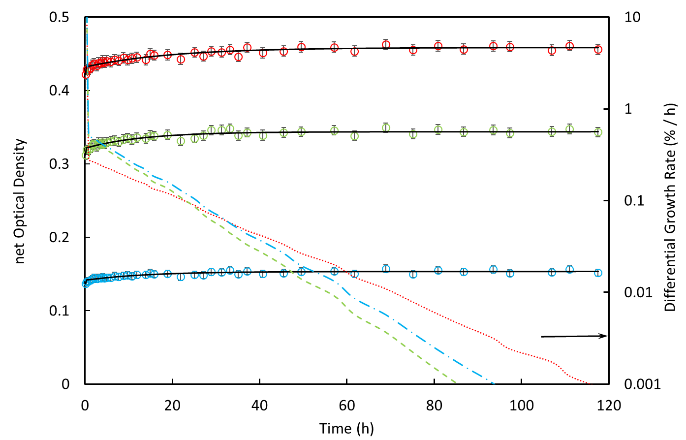 | 10 Gy  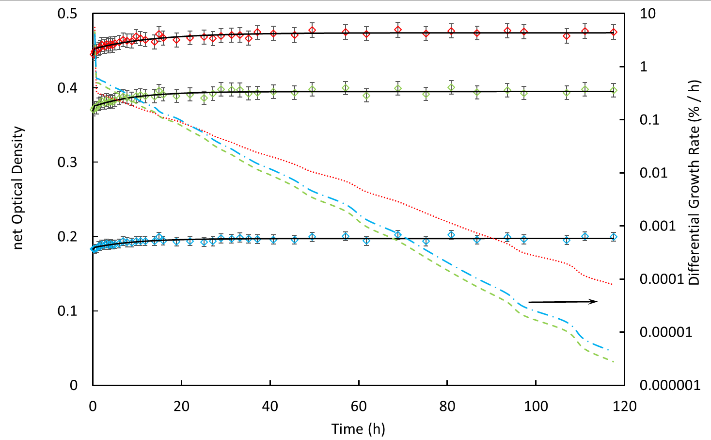 | | |
| Figure S4 Time evolution of netOD for EBT4 (left column) and EBT3 (right column) at 225 MeV proton energy and doses of 1 Gy, 5 Gy, and 10 Gy. The stability of the post-irradiation response is maintained across all doses and color channels. The arrows show the right ordinate for the differential growth rate data for corresponding three colors of EBT4 (left)and EBT3 (right) films. | | | |
